# Spinal Epidural Capillary Hemangioma With Intrathoracic Extension: Case Report and Review of the Literature

**DOI:** 10.7759/cureus.9358

**Published:** 2020-07-23

**Authors:** Sharad Rajpal, Stephen Johs, Callista Zaronias, Robert C Forsythe, Sigita Burneikiene

**Affiliations:** 1 Neurosurgery, Boulder Neurosurgical Associates, Boulder, USA; 2 Surgery, Boulder Community Health, Boulder, USA; 3 Medicine, University of Rochester Medical Center, Rochester, USA; 4 Pathology, Boulder Valley Pathology, Boulder, USA; 5 Neurosurgery, Justin Parker Neurological Institute, Boulder, USA

**Keywords:** spinal epidural capillary hemangioma, intrathoracic extension, case report, video-assisted thoracic surgery

## Abstract

Capillary hemangiomas are hamartomatous congenital vascular malformations that are particularly uncommon in the spinal epidural space, and those with intrathoracic extensions are extremely rare. Although considered benign, capillary hemangiomas can cause rare hemorrhagic complications and risk of spinal cord compression or extension into the neural foramen. Therefore, surgery should be considered even in the absence of neurological symptoms. The literature reports three patients either underwent a partial resection or a complete tumor removal was achieved by accessing the lesion through a posterolateral approach and removing the costotransverse joint.

The patient underwent a same-day, two-staged gross total resection of the tumor via combined posterior right-sided T7-T8 complete facetectomy and extradural mass resection with T7 nerve transection, followed by a posterolateral fusion of the T7-T8 vertebra. Stage 2 consisted of a video-assisted intrathoracic approach for the removal of the remaining tumor.

The two-stage surgical procedure described in our case report allows for complete removal of intrathoracic and intraspinal portions of the mass with less morbidity.

## Introduction

Hemangiomas are one of the most common benign spinal tumors and are usually located in thoracic or lumbar vertebrae, rarely extending into the spinal canal or neural foramina [1,2]. Hemangiomas involving spinal cord and nerves are less frequent and typically occupy the intradural extramedullary space, followed by the intramedullary compartment. Primarily spinal epidural capillary hemangiomas (ECH) are particularly uncommon [3,4].

Capillary hemangiomas are hamartomatous congenital vascular malformations consisting of a cluster of capillaries lined by flattened endothelium with feeding and draining vessels. Although considered benign, capillary hemangiomas can cause rare hemorrhagic complications and risk of spinal cord compression or extension into the neural foramen; surgery should, therefore, be considered even in the absence of neurological symptoms [5-7].

There have been a handful of primarily spinal ECH cases reported in the literature, but cases with intrathoracic extensions are even less common [3,8,9]. Furthermore, our case is important because we report a two-stage procedure removing the intrathoracic and spinal portions of the mass.

## Case presentation

**Figure 1:**
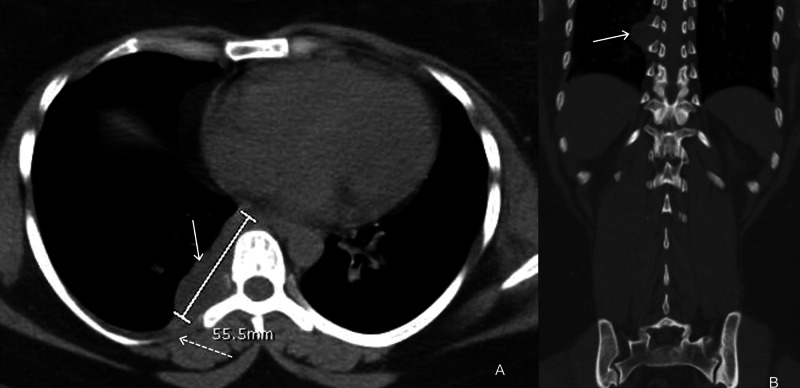
CT scan of the chest, axial (A) and coronal (B) images without contrast show a large 5.5 cm insinuating paravertebral mass (arrow) at the T7 and T8 vertebral bodies with smooth remodeling of the undersurface of the right seventh costovertebral junction (dashed arrow).

A 29-year-old female patient was admitted to the hospital for septic shock with a urinary tract infection, pyelonephritis, and nephrolithiasis. During the hospital admission, the patient underwent an extensive workup, including a CT scan without contrast that showed a right-sided paraspinal mass at the T7 level (Figure 1). 

**Figure 2:**
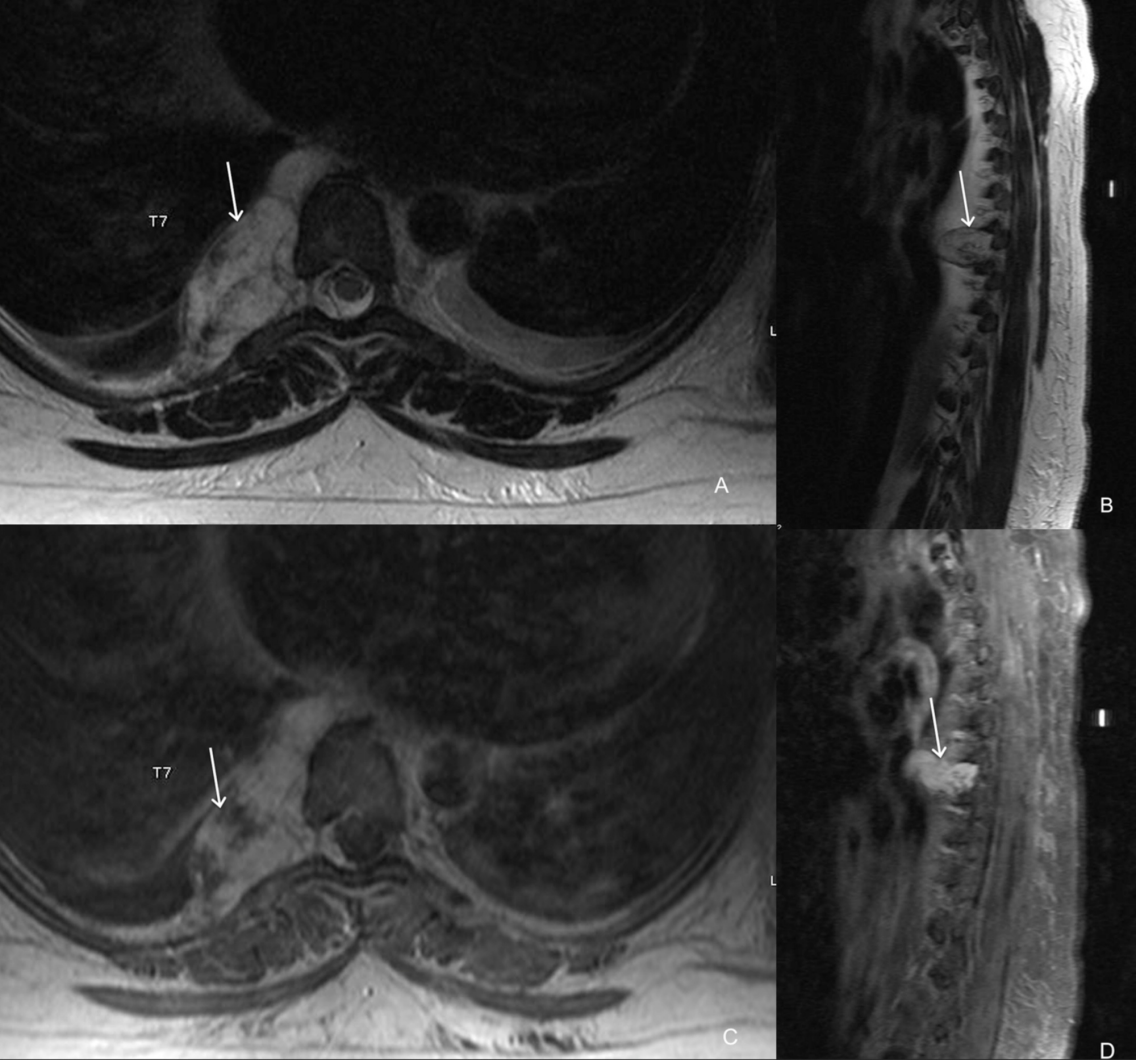
MRI of the thoracic spine, T2-weighted axial (A), sagittal (B) and contrast-enhanced T1-weighted axial (C) and sagittal (D) views demonstrate an avidly enhancing mass (arrow) extending through the right neural foramen and into the right lateral epidural space mildly indenting the right lateral thecal sac. The central canal and left neural foramina are widely patent.

MRI of the thoracic spine with and without contrast demonstrated a 6.2 x 3.1 x 2.1 cm well-circumscribed solid and avidly enhancing right paraspinal T2-hyperintense lesion at the T7 level with extension into the right neural foramen, and into the dorsal and ventral epidural spaces but with no intradural extension. Additional small hemangiomas were seen in the right lateral aspect of the T9 vertebral body, C7, T7, and L1 vertebrae (Figure 2).

The patient was complaining primarily of flank pain secondary to her urinary and kidney stone issues and denied any numbness, tingling, and back or radicular pain. Motor examination of the upper and lower extremities was normal and full strength with normal patellar reflexes, negative Babinski, and intact sensation throughout.

Operation

Given her young age and the location of the mass with an unknown diagnosis (the patient declined biopsy), the patient elected to proceed with surgical resection. She underwent a same-day, two-staged surgery. Stage 1 included a posterior right-sided T7-T8 complete facetectomy, extradural mass resection with T7 nerve transection, and a posterolateral fusion of the T7-T8 vertebra. Under the general anesthesia, the patient was first positioned prone onto the Jackson table and a mid-thoracic incision was taken down through the dorsolumbar fascia, followed by a subperiosteal dissection out to the transverse processes of T7 and T8. Two pedicle screws were placed on the left at T7 and T8. A right-sided complete T7-T8 facetectomy was completed, the exiting nerve root was completely skeletonized, and the dorsal root ganglion (DRG) identified. There was a vascular tumor circumferentially within the foramina, which was curetted and resected. The nerve root proximal to the DRG was coagulated and cut with microscissors in case any tumor was located within the nerve root itself. The remainder of the tumor was cleaned out from this area and the epidural space. The wound was then closed in a standard manner in multiple layers.

**Figure 3:**
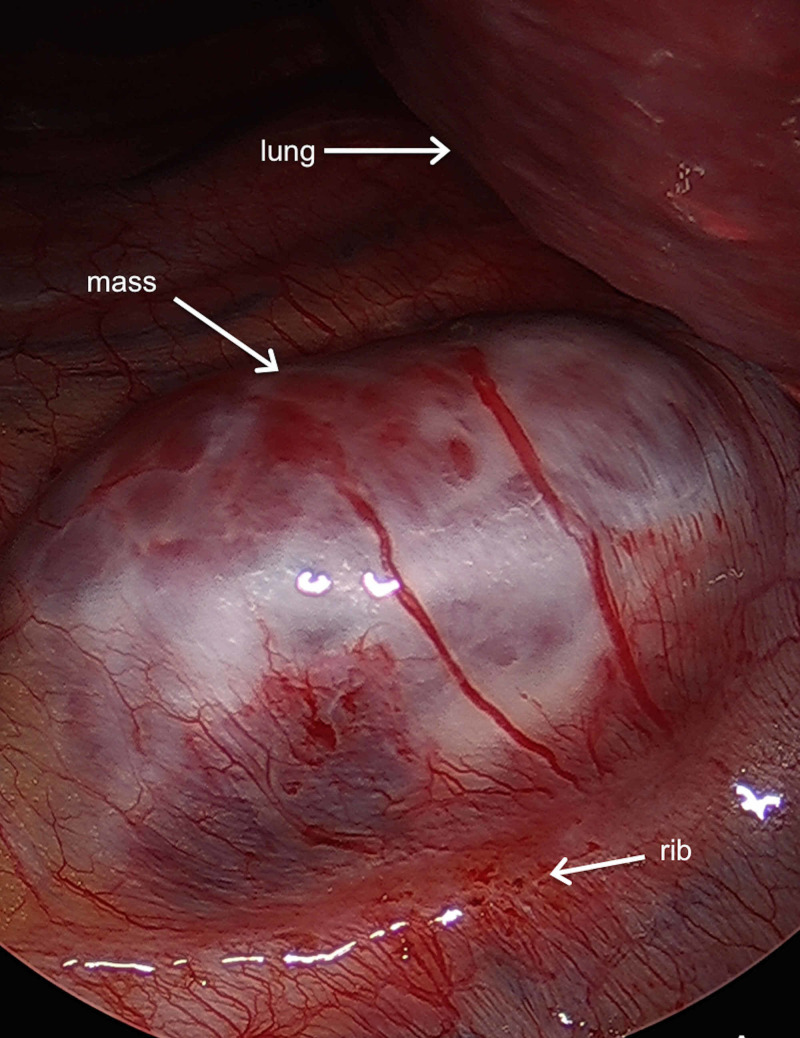
Initial intraoperative viewing of the mass.

Stage 2 was then completed by a general surgeon. The patient was placed into a left lateral decubitus position with slight rotation anteriorly and the chest was entered through the sixth intercostal space through the serratus muscle immediately at the lateral aspect of the inframammary fold. A 15-mm port was placed, and a 30-degree endoscope in the pleural space was introduced. After the lung was deflated, additional 5-mm ports were placed posterior of the eighth intercostal space below the tip of the scapula, at the seventh intercostal space posterior the axillary line, and an additional between the two. The lung was retracted cephalad and the inferior pulmonary ligament was incised and dissected up to the inferior pulmonary vein, providing sufficient visualization of the tumor, which was immediately at the level of the inferior pulmonary vein posteriorly (Figure 3, Video 1).

**Video 1:** Tumor removal.

**Figure 4:**
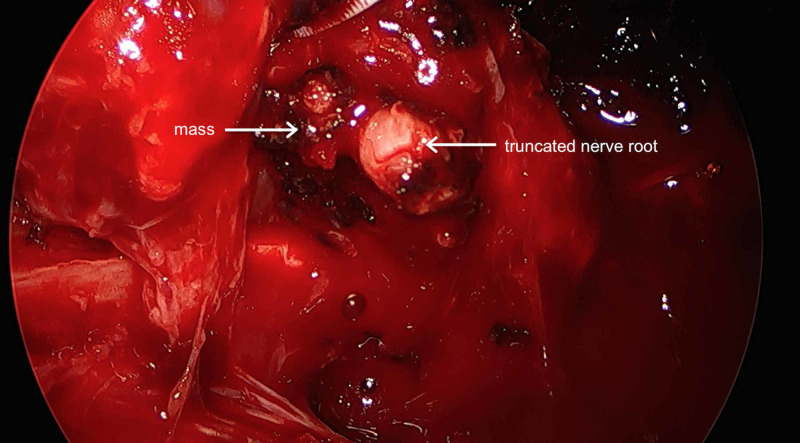
Interval intraoperative viewing of the mass.

The tumor arose over the intercostal space between the seventh and eighth ribs with some overlying neovascularization. The pleura was incised inferiorly, and the dissection was carried up to the posterolateral aspect of the tumor furthest from the spine. The cautery was used to dissect and divide the intercostal nerve allowing the dissection to continue anteriorly and dispatching the intercostal artery and vein laterally. The dissection was carried out circumferentially along the cephalad aspect of the tumor down to the rib, off of the eighth rib periosteum and the intercostal space. The tumor overlapped the inferior aspect of the seventh rib and appeared to derive much of its blood supply from the seventh intercostal artery. Once the tumor had been liberated from the intercostal muscle, it was left attached by the posterior dissection of the nerve root divided earlier (Figure 4).

**Figure 5:**
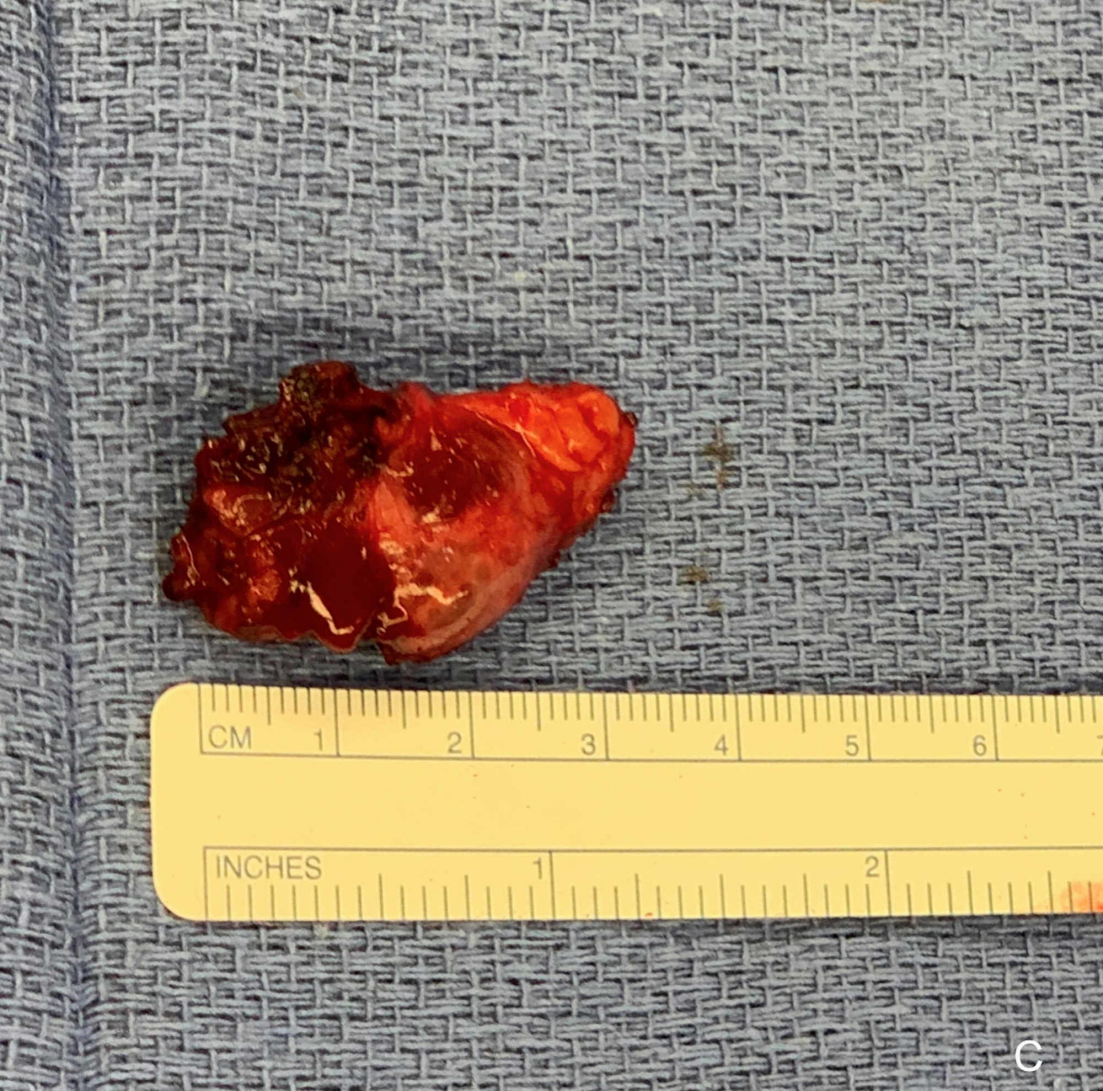
Resected tumor.

The tumor was retrieved using the 15-mm port site (Figure 5).

**Figure 6:**
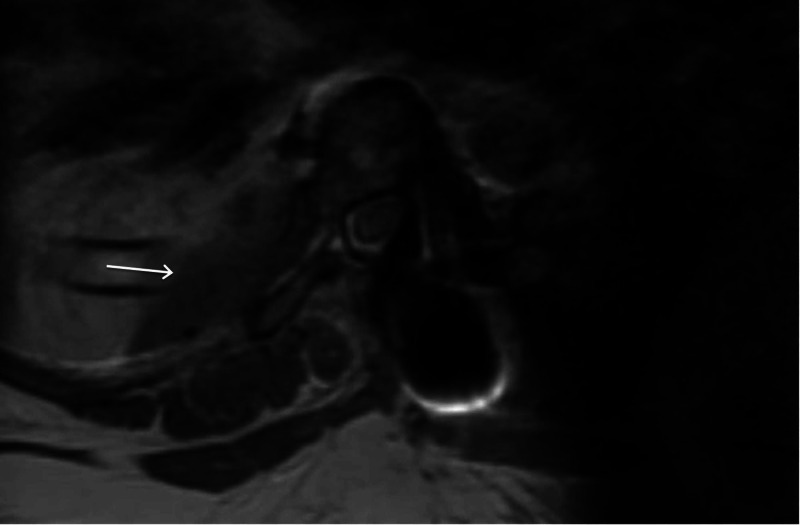
Postoperative MRI of the thoracic spine, contrast-enhanced T2-weighted axial view shows interval resection of previous right T7-T8 paraspinal and neuroforaminal mass with no residual enhancing mass seen. And a small fluid collection identified at the right paraspinal operative site (arrow).

Hemostasis was secured and a chest tube was placed. The lung was reinflated, the ports were removed, and the incisions were closed. Postoperative MRI of the thoracic spine with and without contrast showed gross tumor resection (Figure 6).

The patient’s chest tubes were removed, and she was discharged home on postoperative day 4.

Pathological findings

**Figure 7:**
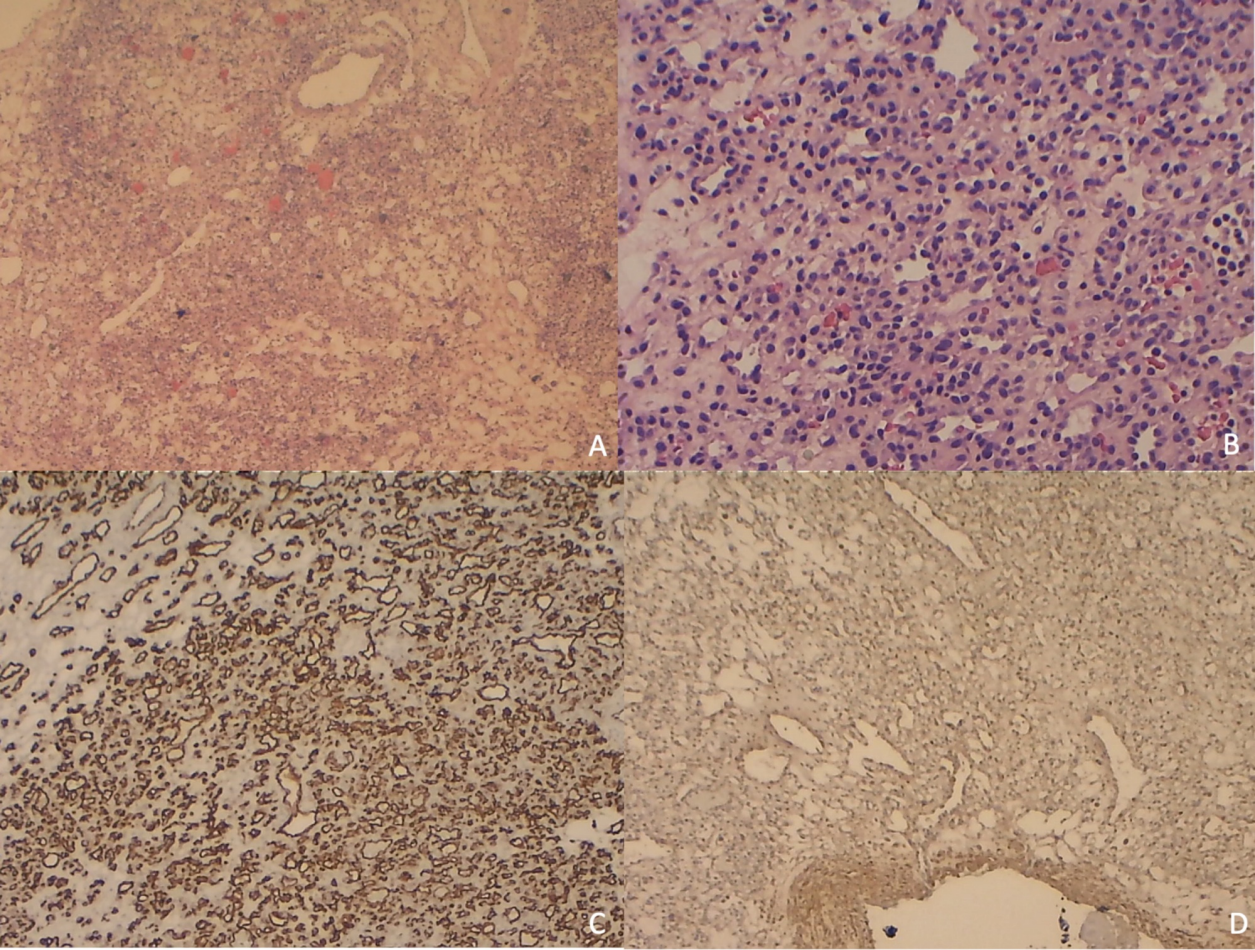
Histology slides: Low-power magnification (A) identified small round cells surrounding small blood vessels and high-power magnification (B) did not demonstrate significant nuclear atypia or mitotic activity; low-power magnification CD34 immunostain was strongly positive (C); low-power magnification smooth muscle actin immunostain was focally positive (D).

The postoperative pathological examination determined the mass to be a hemangioma (Figure 7).

On microscopic examination of the specimens, a well-demarcated and possibly encapsulated population of small round cells surrounding small blood vessels were identified (A). The nucleoli were regular and ovoid, without significant nuclear atypia or mitotic activity (B). Immunohistochemically, CD34 antibody staining was performed and shown to be strongly positive (C). Additionally, an immunostain for smooth muscle actin was performed and was shown to be focally positive, but not diffusely or strongly staining (D). The histopathological information further supported the diagnosis of capillary hemangioma.

## Discussion

Clinical symptoms of ECH depend on the size and location of the mass and usually manifest with radicular symptoms and compressive myelopathy. Contrarily to cavernous hemangiomas, there were no ECH cases reported involving hemorrhages. Although our patient was asymptomatic, it was decided to treat this hemangioma surgically due to her young age and plans to become pregnant. Akhaddar et al. reported on a 19-year-old female patient who developed back pain and progressive gait disturbance in the third trimester of gestation and complete paraplegia in the immediate postpartum period [10]. They hypothesized that increased venous pressure due to the inferior vena cava obstruction, the estrogenic effect on the endothelium leading to the hemangioma size increase, and blood volume during pregnancy distending angiomatous blood vessels might lead to mechanical compression of the spinal cord during pregnancy.

The list of ECH reported cases is presented in Table 1, including the levels involved, tumor extension, and surgery performed. A vast majority of cases (88%) are reported in the thoracic spine, and laminectomy with en bloc resection of the mass is usually performed. Cofano et al. performed embolization to avoid excessive bleeding during surgery; however, the authors did not recommend it because it did not result in devascularization or reduce bleeding due to the compensatory collateral circulation and it could exacerbate the compression in the spinal canal [11].

**Table 1:** Spinal ECH Reported in the Literature. ECH, epidural capillary hemangioma; SC, spinal cord.

	Age/Sex	Location	Extension	Surgery
Gupta et al., 1996 [12]	50 years/M	T8-T10	Dumbbell-shaped ECH with foraminal extension and SC displacement	Total resection
Badinand et al., 2003 [13]	40 years/F	T2-T4	Dumbbell-shaped ECH with foraminal extension	Laminectomy and partial resection
Kang et al., 2006 [3]	56 years/M	T2-T4	Dumbbell-shaped ECH with intrathoracic extensions and SC compression	Laminectomy and resection of the intraspinal and foraminal portions of the mass
Tekin et al., 2008 [14]	56 years/F	L3-L4	ECH with foraminal extension	Laminectomy and total resection
Akhaddar et al., 2010 [10]	19 years/F	T5-T6	ECH with foraminal extension and SC compression	Laminectomy and total resection
Hasan et al., 2011 [7]	57 years/M	T10-T12	ECH with foraminal extension and SC compression	Laminectomy and partial foraminal extension resection
Vassal et al., 2011 [8]	59 years/F	T5-T7	Dumbbell-shaped ECH with foraminal, intrathoracic extensions, and SC compression	Laminectomy, facetectomy, costotransverse joint removal, and total resection
Gencpinar et al., 2014 [6]	17 months/F	T3-T7	ECH with foraminal extension and SC compression	Laminectomy and total resection
Seferi et al., 2014 [15]	58 years/M	T2-T4	ECH with bilateral extension toward intervertebral foramina	Laminectomy and total resection
Garcia-Pallero et al., 2015 [9]	67 years/F	T4-T5	Dumbbell-shaped ECH with foraminal, intrathoracic extensions, and SC compression	Laminectomy, facetectomy, costotransverse joint removal, and total resection
Egu et al., 2016 [16]	60 years/F	L5-S1	ECH with foraminal extension and cauda equina compression	Laminectomy and total resection
Rajeev et al., 2017 [5]	50 years/M	T12-L2	ECH with foraminal extension and severe cauda equina syndrome	Laminectomy and total resection
Brasil et al., 2018 [17]	69 years/F	T9-T10	ECH with SC compression	Laminectomy and total resection
Xu et al., 2018 [4]	57 years/M	T2-T3	ECH with extension toward intervertebral foramina	Laminectomy and total resection
Cofano et al., 2019 [11]	52 years/F	T6-T9	ECH with foraminal extension and SC dislocation	Embolization followed by laminoplasty and total resection
Rajpal et al., 2019	29 years/F	T7-T8	Dumbbell-shaped ECH with intrathoracic and foraminal extensions	Stage 1: Laminectomy, facetectomy, complete resection, and posterolateral fusion. Stage 2: Video-assisted thoracic surgery and resection of the intrathoracic portion

There have been only four ECH with intrathoracic extension reported, including the present case. Kang et al. reported a dumbbell-shaped T2-T4 hemangioma with epidural and intrathoracic extensions [3]. Laminectomy was performed with resection of intraspinal and foraminal portions of the mass, but the authors reported the intrathoracic portion (37 x 41 mm) was inaccessible from the posterior approach and was not removed. Garcia-Pallero et al. and Vassal et al. were able to achieve a complete resection of the intrathoracic portion of the tumor through a posterolateral approach by removing the costotransverse joint and exposing the lesion [8,9].

## Conclusions

This case report adds to the literature by describing an asymptomatic patient with ECH and intrathoracic extension. The two-stage surgical procedure described in our case report allows for video-assisted complete removal of intrathoracic and intraspinal portions of the mass with less morbidity. Previously reported similar cases either described a partial resection or a complete tumor removal by accessing the lesion through a posterolateral approach and removing the costotransverse joint.
